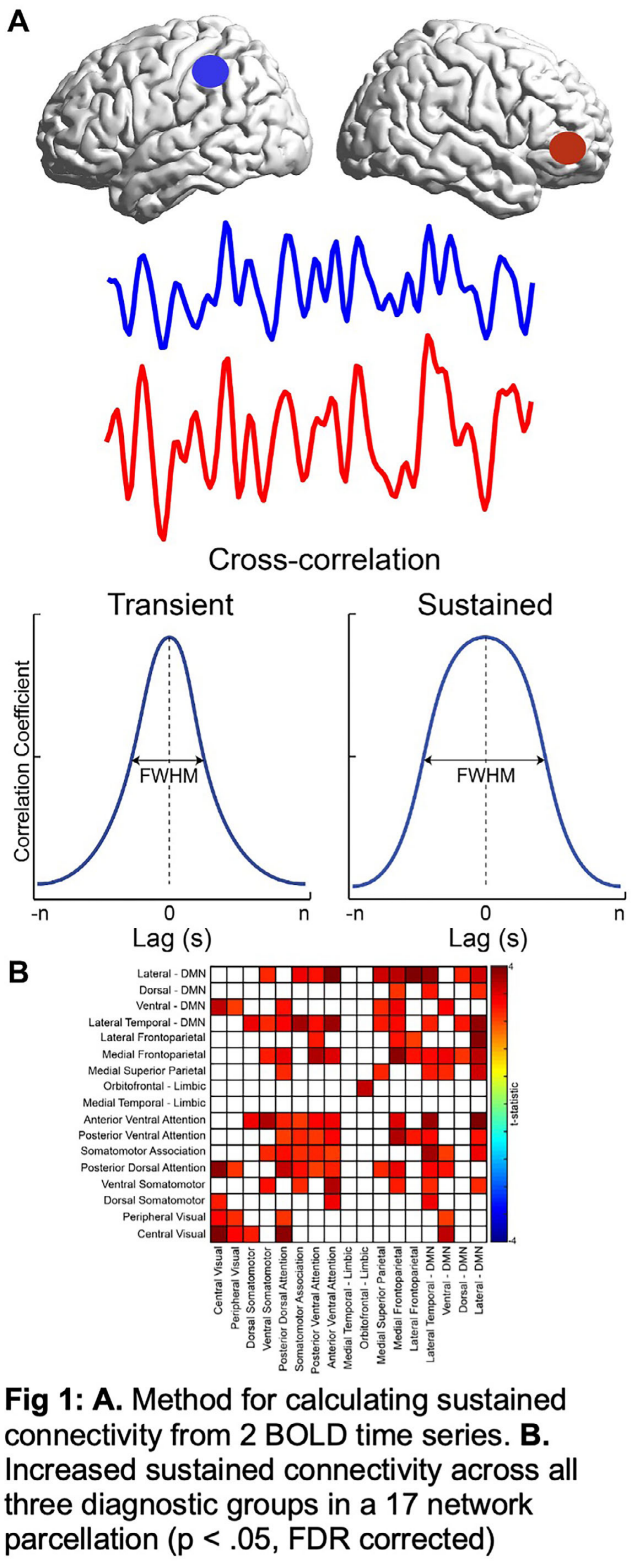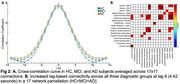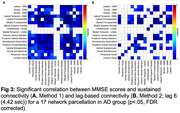# Temporal dynamics of brain connectivity across the dementia spectrum

**DOI:** 10.1002/alz.093207

**Published:** 2025-01-09

**Authors:** Jace B King, Molly B.D. Prigge, Kevin Duff, John M. Hoffman

**Affiliations:** ^1^ University of Utah, Salt Lake City, UT USA; ^2^ Oregon Health & Science University, Portland, OR USA; ^3^ NIA‐Layton Aging & Alzheimer's Disease Research Center, Portland, OR USA; ^4^ Huntsman Cancer Institute, University of Utah, Salt Lake City, UT USA

## Abstract

**Background:**

Traditional functional connectivity is calculated as the correlation between resting‐state fMRI time series from pairs of brain regions or networks. Our innovative method extracts time series information from cross‐correlations curves between brain regions, thus deriving the relative duration of functional connections. These temporal analysis methods provide a means for examining how the timing of brain connections is affected across the dementia spectrum. This study extends previous research efforts into understanding the neural correlates of dementia by applying innovative resting‐state functional connectivity analysis methods to a sample of cognitively intact older adults (HC), individuals with mild cognitive impairment (MCI), and Alzheimer’s disease (AD).

**Method:**

109 participants were included in this analysis (HC: n=47, 29 female, age=72.13±4.68; MCI: n=32, 18 female, age=73.84±5.14; AD: n=30, 19 female, age=77.37±6.45). All study participants were administered the Mini Mental Status Examination (MMSE), and completed MP2RAGE structural imaging and two 15‐minute resting fMRI scans. Cross‐correlation curves were constructed utilizing the Pearson correlation coefficient between pairs of times series for each of the 17 brain networks in the Yeo et al. parcellation. Correlation values were recorded at each of ±20 lags by shifting one of the time points by one volume. Sustained connectivity is derived from the full width at half maximum of the curve (Figure 1A) whereas lag‐based connectivity utilizes the correlation value at each lag. T statistics and p‐values are reported for across‐group connectivity values. Partial correlations were computed between connectivity measures and MMSE scores. All results include controlling for the effects of mean head motion, age, and sex.

**Result:**

Sustained connectivity demonstrated distributed increases across groups (Figure 1B; HC<MCI<AD; pFDR<.05). At zero‐lag, we see decreased connectivity in MCI and AD compared to HC; however, as lag‐based connectivity is increased, we see increases in connectivity corresponding to diagnostic groups (Figure 2; HC<MCI<AD). Across study participants, both sustained and lag‐based connectivity were negatively correlated with MMSE scores (Figure 3; pFDR <.05).

**Conclusion:**

Taken together, these findings suggest that functional connections between brain networks persist longer across disease states and that this longer duration of connectivity may reflect increased cognitive demand related to underlying cognitive processes.